# Hirschsprung Disease and Intestinal Neuronal Dysplasia Type B: Is There a Difference in the Clinical Presentation of These Two Diseases?

**DOI:** 10.7759/cureus.50618

**Published:** 2023-12-16

**Authors:** Anderson Cesar Gonçalves, Bruna Camargo de Oliveira, Mariana Patti Sanches Coelho, Eloa Scalfi Caproni, Giovana Tuccille Comes, Maria Aparecida Marchesan Rodrigues, Erika Veruska Paiva Ortolan, Pedro Luiz Toledo de Arruda Lourenção

**Affiliations:** 1 Department of Surgery - Division of Pediatric Surgery, Botucatu Medical School, São Paulo State University (UNESP), Botucatu, BRA; 2 Surgery, Ouro Verde Hospital, Campinas, BRA; 3 Department of Pathology, Botucatu Medical School, São Paulo State University (UNESP), Botucatu, BRA

**Keywords:** enteric nervous system disorders, children, constipation, intestinal neuronal dysplasia type b, hirschsprung disease

## Abstract

Background: Although the signs and symptoms that comprise the clinical presentation of Hirschsprung disease (HD) and intestinal neuronal dysplasia type B (IND-B) are well established, no studies have specifically compared the clinical characteristics presented by patients with these diseases. We compared the clinical pictures of patients with HD and IND-B at the time of histopathological diagnosis.

Methods: This was a single-center, retrospective, analytical, and comparative study. We included 119 patients aged 0-15 years diagnosed with HD or IND-B. Information from the medical records of these patients was retrieved to obtain demographic and clinical information at the time of diagnosis. The data were compared statistically according to the characteristics of the variables.

Results: Sixty-nine patients (58.0%) were diagnosed with HD, and 50 (42.0%) had IND-B. The HD group had significantly more individuals with symptom onset in the neonatal period (p = 0.001), delayed meconium clearance (p < 0.001), failure to thrive (p = 0.02), and acute complications, such as enterocolitis (p = 0.049) or acute abdominal obstruction (p = 0.031), more commonly requiring emergency surgery (p < 0.001). Patients with IND-B were diagnosed at a significantly older age (p = 0.002). They more commonly had chronic constipation as their main symptom (p = 0.004), with local complications, such as evacuation bleeding (p = 0.007).

Conclusion: There were significant differences between the clinical pictures of patients with HD and IND-B. Knowledge of each disease’s most common signs and symptoms can help direct diagnostic susception and initial management.

## Introduction

Hirschsprung disease (HD) and intestinal neuronal dysplasia type B (IND-B) are two neuromuscular gastrointestinal diseases that appear within the same clinical spectrum, with severe constipation in childhood, accompanied or not by complications, such as acute intestinal obstruction or enterocolitis [1]. The differentiation between these two diseases can only be established by the histopathological analysis of rectal biopsies [2,3].

HD is defined by the absence of ganglion cells in the submucosal and myenteric plexuses of the enteric nervous system, whereas IND-B is characterized by hyperplasia of the submucosal nerve plexuses [1-3]. The treatment for HD is surgery through colorectal pull-through [4]. In contrast, patients diagnosed with IND-B who have no complications must receive conservative treatment with laxatives [5].

Although the signs and symptoms that comprise the clinical presentation of these two diseases are well established in the literature, no studies have specifically compared the clinical characteristics presented in a case series of patients with HD and IND-B [6-9]. Knowledge of the specificities of the clinical presentation of each disease, including demographic variables and the signs and symptoms, can deepen the knowledge about the clinical picture, improving the diagnostic suspicion and the initial management of these two diseases. Therefore, we aimed to compare the clinical and demographic aspects presented by patients with HD and IND-B at the time of their histopathological diagnosis.

## Materials and methods

This single-center, retrospective, analytical, and comparative study was approved by the Research Ethics Committee of the Botucatu Medical School - São Paulo State University (UNESP) under protocol number 55763722.3.0000.5411. We included 119 patients aged 0-15 years diagnosed with HD or IND-B through histopathological analysis of rectal biopsies from 1998 to 2010. The histopathological diagnosis of HD was established based on the absence of ganglion cells in the distal rectum’s submucosal and myenteric nervous plexuses [10,11]. The histopathological diagnosis of IND-B was established according to the morphological criteria proposed by the Frankfurt Consensus (1990) [12]. The patients were stratified into two groups according to the results of the rectal biopsies: the HD group, including 69 patients with a diagnosis of HD, and the IND-B group, including 50 patients diagnosed with IND-B.

Information was retrieved from the patients' medical records. The following data were retrieved and tabulated: 1) clinical and demographic information: sex, gestational age, birth weight, age at symptom onset, and age at diagnosis; 2) information on the clinical picture in the neonatal period: the presence of intestinal symptoms, delay in meconium elimination, and presence of associated malformations; 3) clinical information present at the time of histopathological diagnosis: symptoms related to bowel habits (defecation frequency, episodes of painful or strained defecation, evacuation bleeding, abdominal pain, presence of fecaloma, need for bowel washout, and fecal incontinence), episodes of enterocolitis and acute intestinal obstruction, the need for urgent surgery and failure to thrive; and 4) results of diagnostic screening tests: anorectal manometry and barium enema.

A comparative analysis between groups was performed. Numerical data are presented as the mean values ± standard deviations or median (interquartile deviation), according to the type of data normality distribution previously evaluated using the Kolmogorov-Smirnov test. Proportions are presented as percentages and their respective confidence intervals. Comparisons between the groups were performed using different statistical tests according to the type of variables analyzed. Nominal variables were analyzed using Fisher’s exact test or the chi-square test with Yates correction. Different proportions were compared using a binomial test. Continuous numerical variables with nonparametric distributions were compared using the Mann-Whitney U test, and those with parametric distributions were compared using Student's t test. Relationships and differences were considered statistically significant at p < 0.05. Analysis was performed using SPSS v. 22.0 (IBM Corp, Armonk, NY, USA). The results were graphically summarized using a Venn diagram built using the Adobe CreativeCloud ® tool.

## Results

This study included 119 patients, of whom 88 (74.0%) were males and 31 (26.0%) were females. The median age at symptom onset was 1 (15.5) day, and 76.8% of patients had symptoms in the neonatal period. The median age at the time of histopathological diagnosis was 83 (1,083) days. Sixty-nine (58.0%) patients were diagnosed with HD, and 50 (42.0%) were diagnosed with IND-B, thus composing the two comparison groups (HD group and IND-B group).

Comparison between the HD and IND-B groups

Fifty-three (76.8%) patients with HD and 35 (70.0%) patients with IND-B were males. There was no significant difference in the distribution between sex (p = 0.532; chi-square test) or age at symptom onset (HD: 0 (6.5) days vs IND-B: 2.5 (90) days; p = 0.144; Mann-Whitney U test). However, symptom onset in the neonatal period was significantly more prevalent (p = 0.03; chi-squared test) among patients with HD (86.3%) than among patients with IND-B (66.7%).

The comparison between patients in both groups regarding clinical characteristics presented in the neonatal period is shown in Table 1. Delayed meconium elimination (p < 0.001) and the presence of intestinal symptoms (p = 0.001) were associated with the diagnosis of HD.

**Table 1:** Comparison of clinical characteristics presented by patients with HD and IND-B in the neonatal period. n/T: number of individuals who had a specific clinical characteristic/total number of patients included (the number of patients included in each analysis varied because some information from clinical history were unavailable in certain patients); %: percentage distribution; 95% CI: 95% confidence interval; * p-value associated with Fisher's exact test; # p-value associated with chi-square test.

Clinical characteristics	HD Group			IND-B Group			p
	n/T	%	CI 95%	n/T	%	CI 95%	
Low Birth Weight	4/28	14.3	5.7 – 31.5	8/48	16.7	8.7 – 29.6	1.00 *
Preterm birth	4/32	12.5	5.0 – 28.1	10/48	20.8	11.7 – 34.3	0.385 *
Presence of intestinal symptoms	30/41	73.2	58.1 – 84.3	16/48	33.3	21.7 – 47.5	<0.001 ^#^
Presence of associated malformations	6/40	15.0	7.1 – 29.1	3/48	6.3	2.2 – 16.8	0.289 *
Delay in meconium elimination	36/46	78.3	64.4 – 87.7	18/41	43.9	29.9 – 59.0	0.001 ^#^

Patients with IND-B were significantly older at diagnosis than those in the HD group (HD: 50 (275) days vs IND-B: 365 (1,417) days; p = 0.002; Mann-Whitney U test). At the time of diagnosis (Table 2), most patients with HD presented failure to thrive (p = 0.02) and a history of previous episodes of enterocolitis (p = 0.049). In contrast, evacuation bleeding was more common in patients with IND-B (p = 0.007). There was no significant difference in the maximum number of days without defecation (HD: 9.62 ± 9.26 days vs IND-B: 10.87 ± 8.09 days; p = 0.508; t-test).

**Table 2:** Comparison of clinical characteristics presented by patients with HD and IND-B at diagnosis. n/T: number of individuals who had a specific clinical characteristic/total number of patients included (the number of patients included in each analysis varied because some information from clinical were unavailable in certain patients); %: percentage distribution; 95% CI: 95% confidence interval; * p-value associated with Fisher's exact test; # p-value associated with chi-square test.

Clinical characteristics	HD Group			IND-B Group			p
	n/T	%	CI 95%	n/T	%	CI 95%	
Abdominal distension	59/60	98.3	91.1 – 99.7	43/48	89.6	77.8 – 95.5	0.086 *
Abdominal pain	34/45	75.6	61.3 – 85.8	39/48	81.2	68.1 – 89.8	0.504 ^#^
Evacuation bleeding	5/41	12.2	5.3 – 25.5	18/41	43.9	29.9 – 59.0	0.007 *
Presence of fecaloma	11/43	25.6	14.9 – 40.2	17/48	35.4	23.4 – 49.6	0.310 ^#^
Need for bowel washout	30/46	65.2	50.8 – 77.3	37/48	77.1	63.5 – 86.7	0.203 ^#^
Episodes of enterocolitis	12/41	29.3	17.6 – 44.5	6/48	12.5	5.9 – 24.7	0.049 ^#^
Failure to thrive	15/28	53.6	35.8 – 70.5	13/48	27.1	16.6 – 41.0	0.020 ^#^

Regarding the main clinical picture presented at diagnosis (Table 3), constipation was more common in patients with IND-B (p = 0.004), and acute abdominal obstruction was more common in patients with HD (p = 0.031). Because of this, the need for urgent surgery was significantly higher (p < 0.001; chi-square test) in the HD group (62.0 [48.1%-74.1%]) than in the IND-B group (22.9 [13.3%-36.5%]).

**Table 3:** Comparison of the main clinical picture presented by patients with HD and IND-B at diagnosis. n/T: number of individuals who had a specific clinical characteristic/total number of patients included; %: percentage distribution; 95% CI: 95% confidence interval; # p-value associated with the chi-square test.

Main clinical picture at diagnosis	HD Group			IND-B Group			p
	n/T	%	CI 95%	n/T	%	CI 95%	
Abdominal distension	12/51	23.5	14.3 – 36.8	6/48	12.5	5.9 – 24.7	0.155 ^#^
Constipation	25/51	49.0	35.9 – 62.3	37/48	77.1	63.5 – 86.7	0.004 ^#^
Acute abdominal obstruction	14/51	27.5	17.1 – 40.9	5/48	10.4	4.5 – 22.2	0.031 ^#^

Complementary diagnostic screening tests (Table 4) showed that the absence of the rectoanal inhibitory reflex on anorectal manometry was more common in HD patients (p = 0.028).

**Table 4:** Comparison of results of diagnostic screening tests in patients with HD and IND-B. n/T: number of individuals who had a specific clinical characteristic/total number of patients included (the number of patients included in each analysis varied because some information from complementary tests were unavailable in certain patients); %: percentage distribution; 95% CI: 95% confidence interval; * p-value associated with Fisher's exact test; # p-value associated with chi-square test.

Results of diagnostic screening tests	HD Group			IND-B Group			p
	n/T	%	CI 95%	n/T	%	CI 95%	
Presence of tranzition zone on barium enema	23/32	71,9	54.6 – 84.4	6/11	54.5	28.0 – 78.7	0.456 *
Absence of the rectoanal inhibitory reflex on anorectal manometry	43/48	89.6	77.8 – 95.5	30/48	62.5	48.4 – 74.8	0.028 ^#^

**Figure 1:**
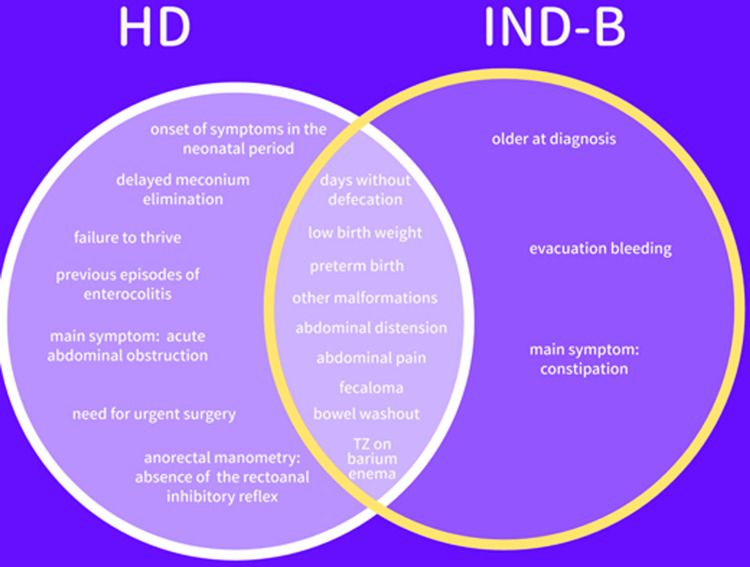
Distribution of the main clinical and demographic aspects found in comparing patients with HD and IND-B.

The Venn diagram in Figure 1 summarizes the distribution of the main clinical and demographic aspects found in comparing patients with HD and IND-B.

## Discussion

Our study observed a male predominance in patients with HD and IND-B. The prevalence of males in HD is well-defined, with proportions ranging from 3:1 to 4:1 [12]. This ratio is influenced by the extent of the aganglio­nic segment, with percentages ranging from 1:1 to 2:1 in long forms and 0.8:1 in total colonic aganglionosis [13,14].

The number of patients born prematurely or with low birth weight was limited and did not differ between the two diseases. HD and IND-B typically affect full-term infants with adequate birth weight [9,13,15]. The onset of symptoms in the neonatal period, including delayed meconium passage, was more common in patients with HD than in those with IND-B. Clinical manifestations of HD depend directly on the extent and degree of spasticity in the aganglionic segment [16]. In most cases, symptoms appear in the first few days of life, with changes in bowel habits, failure to thrive, flatulence, and vomiting. Up to 90% of cases are present in the neonatal period and are characterized by intestinal obstructions [13,17]. Delay in meconium passage in the first 24 to 48 hours of life is reported in up to 90% of patients with HD [13,15]. A minority of patients do not have symptoms during the neonatal period and experience severe constipation throughout childhood [13]. Regarding IND-B, some studies have shown that most patients have symptoms in the first year of life but not in the neonatal period or subsequent months [7,18]. Delayed meconium passage can also be observed; however, it is less common than in HD [7].

More severe conditions due to acute or chronic complications were higher in patients with HD than in those with IND-B. Patients with HD showed more failure to thrive, episodes of enterocolitis, and acute intestinal obstruction, requiring a more significant number of urgent surgical approaches. Enterocolitis associated with HD is the most severe clinical complication related to the disease and can evolve into dehydration, sepsis, and death [13,19]. Its incidence in HD ranges from 12% to 58% of patients and may occur before or after surgical treatment. This complication can reach mortality rates of 1%-10%, more frequently in newborns, before definitive surgery [19,20]. Clinical pictures compatible with a diagnosis of enterocolitis in patients with IND-B have been described in some cases; however, they are less common in those with HD [7,8]. HD represents 20%-25% of cases of intestinal obstruction in the neonatal period [21]. Acute obstructive symptoms can also occur in older children. Many of these patients do not respond positively to the initial measures of clinical treatment and require urgent surgical approaches with temporary colostomies [22]. Evolution to acute intestinal obstruction can also occur in patients with IND-B; however, it is less common than in HD. It is the most frequently reported complication in patients with IND-B and is often the determining factor for surgical treatment [5].

In contrast, chronic constipation as the main symptom was more common in patients with IND-B than in those with HD. Constipation of varying severity is the most common clinical condition in patients with IND-B [23]. Most of these cases evolve insidiously, with little response to conventional treatments for constipation and without acute complications. This corroborates that we observed that the age at diagnosis was significantly higher in patients with IND-B than in those with HD. Less severe symptoms, with chronic evolution and without acute complications, may lead to a delay in referral to specialized services and, consequently, to a delay in diagnosis. Publications in the last two decades have highlighted the increasing number of IND-B cases diagnosed in adults, some with symptoms of constipation since childhood [24-26]. In our study, there was a significant difference in bleeding evacuation among the chronic symptoms related to defecation, which was more common in patients with IND-B than in those with HD. This type of bleeding is associated with the evacuation of bulky and hardened stools that lead to injuries to the perianal mucosa and can be considered a sign of local severity.

Among the complementary tests usually used for the initial diagnostic investigation of HD, we observed a significant difference in the absence of the rectoanal inhibitory reflex on anorectal manometry, which was more common in patients with HD than in those with IND-B. Anorectal manometry is considered an initial screening method in the investigation of HD [11], with specificity rates of 94.2%, a sensitivity of 88.4%, and false-positive results that range from 0% to 62%. If the result is the absence of the recto-anal inhibitory reflex, the child should undergo a rectal biopsy for a diagnostic conclusion [11]. However, using anorectal manometry in the diagnostic workup for IND-B remains controversial, with variable results [7,23,27,28]. It should be noted that in our series, the absence of reflex occurred in 62.5% of the patients with IND-B. During the diagnostic workup for HD, this result led to rectal biopsies, which led to the diagnosis of IND-B.

Identifying the transition zone between the spastic-aganglionic segment and the dilated colon in the barium enema is another method used in the diagnostic screening for HD, with sensitivity and specificity rates of 73% and 90%, respectively [11]. Although 75% of neonates with HD present with a transition zone, the absence of this sign does not exclude the possibility of aganglionosis [29]. However, using these radiological findings in the initial diagnostic investigation of patients with IND-B remains controversial. Most patients with IND-B do not exhibit barium enema-specific radiological features. Like most patients with intestinal constipation, there is usually an increase in the caliber of the rectum and sigmoid. A minority of patients may present with conical colon dilation similar to that observed in HD [7,27]. In our study, there was no significant difference in identifying the transitional zone between patients with HD and those with IND-B. However, it should be noted that almost half of the patients with IND-B did not present with this finding on barium enema. Considering that barium enema is usually focused on diagnostic screening for HD and that identifying the transition zone is the criterion for performing a rectal biopsy, the absence of this finding could lead to delays or errors in diagnosing patients with IND-B.

This study has limitations, such as its retrospective design based on clinical information obtained from a single center medical records. Specific information from clinical history and complementary tests were unavailable; therefore, the number of patients included in each comparative analysis varied. In addition, most barium enema radiographic images were not available for reanalysis. Therefore, this study was performed using only examination reports. However, this is the first study to specifically compare the clinical picture of patients with HD and those with IND-B. The number of patients included can also be considered a strength in this study because it dealt with two rare diseases.

## Conclusions

Although HD and IND-B are part of the same clinical spectrum and the differentiation between these two diseases depends on the histopathological analysis of rectal biopsies, a more profound knowledge of particularities about the clinical presentation of each of these diseases can help to direct the diagnostic suspicion and the initial management. Our study identified two different clinical pictures, one for each disease, based on significant differences in the comparative analyses. In most cases, patients with HD experienced symptoms in the neonatal period, with delayed meconium passage. In addition, they had more severe conditions characterized by acute complications such as enterocolitis and acute abdominal obstruction and chronic complications such as failure to thrive. Therefore, they commonly require urgent surgery. In most cases, patients with IND-B were diagnosed late with chronic and insidious conditions of intestinal constipation, refractory to conventional treatment. The complications presented by these patients were limited and related to evacuation symptoms such as evacuation bleeding.
